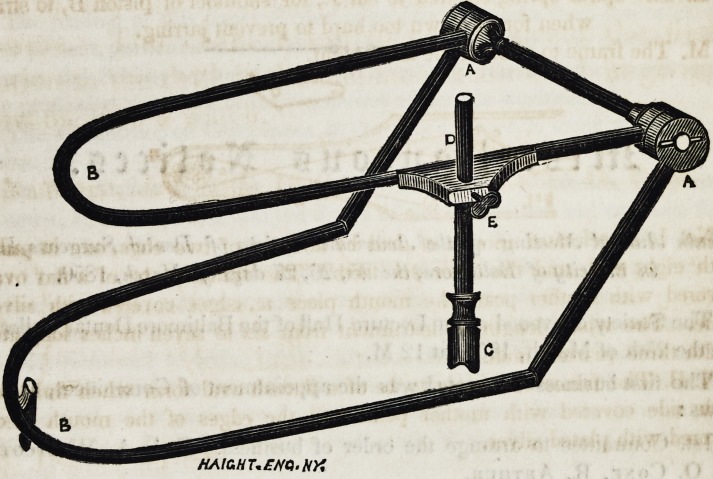# Evans' Antagonizing Instrument

**Published:** 1850-04

**Authors:** John D. Chevalier


### ARTICLE IX.

### Evans' *Antagonizing Instrument.*

New York, *May* 11*th*, 1850.

*Dear Sir:*—Having some time since been furnished by Dr. Parmly, with one of Dr. Evans' instruments for antagonizing teeth, as a model to manufacture by, I had a wood cut of it made for the purpose of illustrating its construction in my circulars.

In looking over the October Number of the Journal of Dental Science, I observed a favorable notice of this instrument, from the pen of the Baltimore Editor, and supposing that an engraving and description of the modus operandi of this instrument, would be acceptable to the readers of the American Journal and Library of Dental Science, I take the liberty of sending you the wood cut, hoping that you will give it a place in the next number of your valuable journal.

With sentiments of the highest regard,

TO DR. C. A. HARRIS. JOHN D. CHEVALIER.

The manner of using this instrument as described to me by Dr. Jahial Parmly, is as follows:

First, strike up the plate in the usual way, and fit it to the mouth, after which open the instrument, (it having hinges at A, A,) lay it over a piece of paper on your work bench, then build up with plaster at B, on the curved extremity of the lower bar, if for upper teeth, and vice versa, if for lower, put your wax impression on the plaster, having previously filled the spaces for the teeth in the wax with plaster, in order to get them perfect, let your patient bite into a piece of wax to get the desired expression of the face, mark the centre with a pointed instrument, remove the first wax from your plaster, put the second with the plate adhering to it in its place, bring the two bars or jaws of the instrument together, guage them with the rod D, allowing the end C, to rest on the bar B, set it with the screw E, build up with plaster as before, trim it and you have a perfect articulating cast. J. D. C.

## Figures and Tables

**Figure f1:**